# *N*-[2-(4-Chloro­phen­yl)propano­yl]-1-methyl­bornane-10,2-sultam

**DOI:** 10.1107/S1600536808021673

**Published:** 2008-07-19

**Authors:** Wen-Chang Lu, Guang-Ao Yu, Xiu-Fang Cao, Shan Jin, Sheng-Hua Liu

**Affiliations:** a Key Laboratory of Pesticides and Chemical Biology, College of Chemistry, Central China Normal University, Wuhan 430079, People’s Republic of China

## Abstract

In the mol­ecular structure of the title compound, C_20_H_26_ClNO_3_S, the six-membered ring of the bornane unit shows a boat conformation, while the five-membered ring of the sultam unit adopts a twist conformation. In the crystal structure, mol­ecules are connected by inter­molecular C—H⋯O hydrogen bonds into a chain running along the *b* axis. Intramolecular C—H⋯O and C—H⋯N hydrogen bonds are also present.

## Related literature

For related literature, see: Lu *et al.* (2008 ▶); Oppolzer (1989 ▶, 1990 ▶).
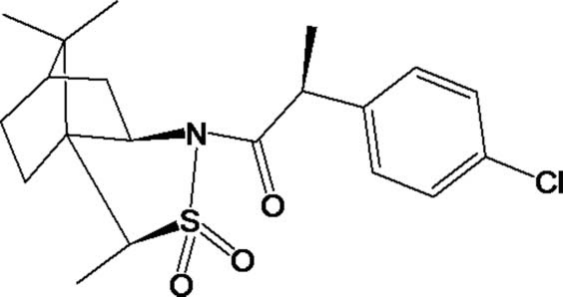


## Experimental

### 

#### Crystal data

C_20_H_26_ClNO_3_S
*M* *_r_* = 395.93
Monoclinic, 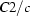

*a* = 24.6517 (10) Å
*b* = 7.6430 (3) Å
*c* = 22.1608 (9) Å
β = 109.477 (1)°
*V* = 3936.4 (3) Å^3^
*Z* = 8
Mo *K*α radiation
μ = 0.32 mm^−1^
*T* = 294 (2) K
0.20 × 0.10 × 0.10 mm

#### Data collection

Bruker SMART CCD area-detector diffractometer
Absorption correction: none
13035 measured reflections
4301 independent reflections
3266 reflections with *I* > 2σ(*I*)
*R*_int_ = 0.044

#### Refinement

*R*[*F* ^2^ > 2σ(*F* ^2^)] = 0.053
*wR*(*F* ^2^) = 0.140
*S* = 1.01
4301 reflections
239 parameters
H-atom parameters constrained
Δρ_max_ = 0.45 e Å^−3^
Δρ_min_ = −0.24 e Å^−3^

### 

Data collection: *SMART* (Bruker, 2001 ▶); cell refinement: *SAINT-Plus* (Bruker, 2001 ▶); data reduction: *SAINT-Plus*; program(s) used to solve structure: *SHELXS97* (Sheldrick, 2008 ▶); program(s) used to refine structure: *SHELXL97* (Sheldrick, 2008 ▶); molecular graphics: *SHELXTL* (Sheldrick, 2008 ▶); software used to prepare material for publication: *SHELXTL*.

## Supplementary Material

Crystal structure: contains datablocks I, global. DOI: 10.1107/S1600536808021673/pv2086sup1.cif

Structure factors: contains datablocks I. DOI: 10.1107/S1600536808021673/pv2086Isup2.hkl

Additional supplementary materials:  crystallographic information; 3D view; checkCIF report

## Figures and Tables

**Table 1:** Hydrogen-bond geometry (Å, °)

*D*—H⋯*A*	*D*—H	H⋯*A*	*D*⋯*A*	*D*—H⋯*A*
C20—H20⋯O3	0.93	2.56	3.038 (3)	112
C13—H13⋯O1	0.98	2.49	3.278 (2)	137
C9—H9*C*⋯N1	0.96	2.52	3.098 (3)	118
C10—H10⋯O3^i^	0.98	2.54	3.191 (2)	124

Symmetry code: (i) 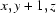.

**Fig. 1.**
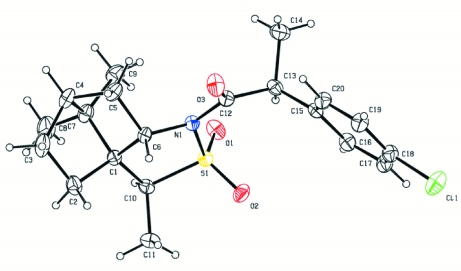
A perspective drawing of the structure of the title compound with displacement ellipsoids plotted at 50% probability level.

## supplementary crystallographic information

### Comment

Pioneering work of Oppolzer (1990) has resulted in the development of bornane[10,2]sultams which serve as popular and widely used chiral auxiliaries in asymmetric synthesis. The resulting asymmetric induction using these auxiliaries are high in carbon-carbon bond formation such as alkylation (Oppolzer, 1989), and we have focused our attention on this field (Lu *et al.*,  2008). In this paper, we present X-ray crystallographic analysis of the title compound, (I).

The structure of title compound (I) is different from that of the reported compound (Lu *et al.*,  2008) wherein a proton is substituted by methyl on C10. In (I), the six-member ring of sultam shows a boat conformation (Fig. 1). The planes constructed by C1/C2/C3/C4 and C1/C6/C5/C4 form a dihedral angle of 110.7 (1)°. The C7/C8/C9 plane makes dihedral angles of 90.3 (1)° and 86.5 (2)°, respectively, with C1/C2/C3/C4 and C1/C6/C5/C4 planes. Molecules are linked by intermolecular C—H···O type hydrogen bonds into a one-dimensional chain; intramolecular interactions of the types C—H···O and C—H···N are also present (details are given in Table 1).

### Experimental

n-BuLi (4.8 ml, 12.0 mmol) in hexane (25.0 ml) was added over 30 min to a THF (25.0 ml) solution of (+)-*N*-[2-(4-chlorophenyl)-ethanoyl]bornane-10,2-sultam (1.84 g, 5.0 mmol) at 193 K. After stirring the mixture at 193 K for 1 h, iodomethane (3.2 ml, 51.4 mmol) in hexamethylphosphorous triamide (4.5 ml, 24.6 mmol) was added and then stirred at 193 K for 3 h. The solution was slowly warmed up to room temperature, quenched with water and extraxted by Et_2_O to afford a crude product. Single crystals appropriate for data collection were obtained by slow evaporation of a dichloromethane solution at 293 K.

### Refinement

All H atoms were constrained to an ideal geometry with C—H = 0.93, 0.96, 0.97 and 0.98 Å for the aromatic, CH_3_, CH_2_ and CH type H-atoms, respectively, and *U*_iso_(H) = 1.5Ueq(methyl C) or 1.2*U*eq(the rest C).

### Crystal data

**Table:**

C_20_H_26_ClNO_3_S	*F*_000_ = 1680
*M**_r_* = 395.93	*D*_x_ = 1.336 Mg m^−^^3^
Monoclinic, *C*2/*c*	Mo *K*α radiation λ = 0.71073 Å
Hall symbol: -C 2yc	Cell parameters from 3899 reflections
*a* = 24.6517 (10) Å	θ = 2.9–26.1º
*b* = 7.6430 (3) Å	µ = 0.32 mm^−^^1^
*c* = 22.1608 (9) Å	*T* = 294 (2) K
β = 109.4770 (10)º	Block, colorless
*V* = 3936.4 (3) Å^3^	0.20 × 0.10 × 0.10 mm
*Z* = 8	

### Data collection

**Table:**

Bruker SMART CCD area-detector diffractometer	3266 reflections with *I* > 2σ(*I*)
Radiation source: fine-focus sealed tube	*R*_int_ = 0.044
Monochromator: graphite	θ_max_ = 27.0º
*T* = 294(2) K	θ_min_ = 1.8º
φ and ω scans	*h* = −31→30
Absorption correction: none	*k* = −9→6
13035 measured reflections	*l* = −28→28
4301 independent reflections	

### Refinement

**Table:**

Refinement on *F*^2^	Secondary atom site location: difference Fourier map
Least-squares matrix: full	Hydrogen site location: inferred from neighbouring sites
*R*[*F*^2^ > 2σ(*F*^2^)] = 0.053	H-atom parameters constrained
*wR*(*F*^2^) = 0.140	*w* = 1/[σ^2^(*F*_o_^2^) + (0.082*P*)^2^] where *P* = (*F*_o_^2^ + 2*F*_c_^2^)/3
*S* = 1.01	(Δ/σ)_max_ < 0.001
4301 reflections	Δρ_max_ = 0.45 e Å^−^^3^
239 parameters	Δρ_min_ = −0.24 e Å^−^^3^
Primary atom site location: structure-invariant direct methods	Extinction correction: none

### Special details

**Table:**

Geometry. All e.s.d.'s (except the e.s.d. in the dihedral angle between two l.s. planes) are estimated using the full covariance matrix. The cell e.s.d.'s are taken into account individually in the estimation of e.s.d.'s in distances, angles and torsion angles; correlations between e.s.d.'s in cell parameters are only used when they are defined by crystal symmetry. An approximate (isotropic) treatment of cell e.s.d.'s is used for estimating e.s.d.'s involving l.s. planes.
Refinement. Refinement of *F*^2^ against ALL reflections. The weighted *R*-factor *wR* and goodness of fit *S* are based on *F*^2^, conventional *R*-factors *R* are based on *F*, with *F* set to zero for negative *F*^2^. The threshold expression of *F*^2^ > σ(*F*^2^) is used only for calculating *R*-factors(gt) *etc*. and is not relevant to the choice of reflections for refinement. *R*-factors based on *F*^2^ are statistically about twice as large as those based on *F*, and *R*- factors based on ALL data will be even larger.

### Fractional atomic coordinates and isotropic or equivalent isotropic displacement parameters (Å^2^)

**Table:**

	*x*	*y*	*z*	*U*_iso_*/*U*_eq_	
C1	0.11339 (8)	0.3105 (3)	−0.06399 (9)	0.0407 (5)	
C2	0.11867 (10)	0.3198 (3)	−0.13128 (10)	0.0594 (6)	
H2A	0.1137	0.4387	−0.1474	0.071*	
H2B	0.1558	0.2766	−0.1308	0.071*	
C3	0.06989 (11)	0.2015 (4)	−0.17175 (11)	0.0759 (8)	
H3A	0.0849	0.1021	−0.1884	0.091*	
H3B	0.0434	0.2656	−0.2072	0.091*	
C4	0.04034 (10)	0.1423 (4)	−0.12372 (11)	0.0619 (6)	
H4	0.0007	0.1016	−0.1437	0.074*	
C5	0.08001 (9)	0.0101 (3)	−0.07791 (11)	0.0560 (6)	
H5A	0.0912	−0.0828	−0.1012	0.067*	
H5B	0.0617	−0.0407	−0.0495	0.067*	
C6	0.13243 (8)	0.1241 (3)	−0.04048 (8)	0.0390 (4)	
H6	0.1660	0.0908	−0.0521	0.047*	
C7	0.04633 (9)	0.3049 (3)	−0.08060 (11)	0.0538 (6)	
C8	0.01610 (12)	0.4693 (4)	−0.11566 (14)	0.0815 (9)	
H8A	−0.0248	0.4522	−0.1297	0.122*	
H8B	0.0280	0.4909	−0.1520	0.122*	
H8C	0.0261	0.5677	−0.0872	0.122*	
C9	0.02409 (9)	0.2754 (3)	−0.02455 (12)	0.0634 (6)	
H9A	−0.0171	0.2656	−0.0405	0.095*	
H9B	0.0351	0.3723	0.0045	0.095*	
H9C	0.0404	0.1696	−0.0026	0.095*	
C10	0.14806 (8)	0.4422 (2)	−0.01475 (9)	0.0419 (5)	
H10	0.1229	0.5401	−0.0131	0.050*	
C11	0.20150 (10)	0.5146 (3)	−0.02619 (12)	0.0589 (6)	
H11A	0.2241	0.4196	−0.0333	0.088*	
H11B	0.2239	0.5803	0.0106	0.088*	
H11C	0.1901	0.5896	−0.0631	0.088*	
C12	0.16253 (9)	−0.0273 (3)	0.06472 (10)	0.0444 (5)	
C13	0.17899 (9)	−0.0208 (3)	0.13723 (9)	0.0451 (5)	
H13	0.1768	0.1006	0.1504	0.054*	
C14	0.13447 (11)	−0.1306 (4)	0.15525 (13)	0.0689 (7)	
H14A	0.1346	−0.2482	0.1400	0.103*	
H14B	0.0969	−0.0805	0.1361	0.103*	
H14C	0.1441	−0.1318	0.2009	0.103*	
C15	0.23966 (9)	−0.0879 (3)	0.16938 (9)	0.0419 (5)	
C16	0.28060 (10)	0.0159 (3)	0.21258 (10)	0.0507 (5)	
H16	0.2705	0.1279	0.2213	0.061*	
C17	0.33565 (10)	−0.0420 (3)	0.24289 (10)	0.0558 (6)	
H17	0.3623	0.0291	0.2723	0.067*	
C18	0.35098 (9)	−0.2068 (3)	0.22924 (10)	0.0530 (6)	
C19	0.31159 (10)	−0.3138 (3)	0.18749 (11)	0.0551 (6)	
H19	0.3222	−0.4253	0.1788	0.066*	
C20	0.25609 (10)	−0.2553 (3)	0.15831 (10)	0.0507 (5)	
H20	0.2292	−0.3294	0.1307	0.061*	
Cl1	0.42204 (3)	−0.27529 (11)	0.26487 (4)	0.0863 (3)	
N1	0.14780 (7)	0.1266 (2)	0.03007 (7)	0.0372 (4)	
O1	0.13477 (7)	0.3813 (2)	0.09856 (7)	0.0581 (4)	
O2	0.23002 (7)	0.32702 (19)	0.09180 (8)	0.0600 (4)	
O3	0.15939 (8)	−0.1640 (2)	0.03573 (8)	0.0674 (5)	
S1	0.16878 (2)	0.32760 (6)	0.06102 (2)	0.03981 (17)	

### Atomic displacement parameters (Å^2^)

**Table:**

	*U*^11^	*U*^22^	*U*^33^	*U*^12^	*U*^13^	*U*^23^
C1	0.0384 (10)	0.0434 (12)	0.0414 (10)	0.0058 (8)	0.0147 (8)	0.0016 (8)
C2	0.0620 (15)	0.0737 (17)	0.0449 (12)	0.0133 (12)	0.0208 (11)	0.0074 (11)
C3	0.0673 (16)	0.110 (2)	0.0410 (12)	0.0176 (16)	0.0052 (12)	−0.0073 (13)
C4	0.0411 (12)	0.0788 (17)	0.0534 (13)	0.0024 (11)	−0.0008 (10)	−0.0144 (12)
C5	0.0514 (13)	0.0531 (14)	0.0563 (13)	−0.0049 (11)	0.0084 (10)	−0.0202 (11)
C6	0.0379 (10)	0.0397 (11)	0.0366 (9)	0.0034 (8)	0.0089 (8)	−0.0066 (8)
C7	0.0405 (12)	0.0618 (15)	0.0549 (12)	0.0107 (10)	0.0102 (10)	−0.0027 (11)
C8	0.0630 (16)	0.094 (2)	0.0807 (18)	0.0351 (15)	0.0143 (14)	0.0139 (16)
C9	0.0410 (12)	0.0797 (18)	0.0727 (15)	0.0037 (12)	0.0232 (11)	−0.0082 (13)
C10	0.0465 (11)	0.0315 (10)	0.0502 (11)	0.0040 (8)	0.0194 (9)	0.0046 (8)
C11	0.0618 (14)	0.0520 (14)	0.0708 (15)	−0.0099 (11)	0.0324 (12)	0.0014 (11)
C12	0.0507 (12)	0.0298 (10)	0.0495 (11)	−0.0010 (9)	0.0123 (9)	−0.0026 (9)
C13	0.0590 (13)	0.0325 (11)	0.0457 (11)	0.0000 (9)	0.0202 (10)	0.0020 (8)
C14	0.0620 (15)	0.0672 (17)	0.0843 (18)	−0.0009 (13)	0.0336 (14)	0.0167 (14)
C15	0.0557 (12)	0.0372 (11)	0.0364 (9)	−0.0032 (9)	0.0202 (9)	0.0022 (8)
C16	0.0706 (15)	0.0392 (12)	0.0425 (11)	−0.0026 (11)	0.0191 (10)	−0.0092 (9)
C17	0.0621 (14)	0.0539 (14)	0.0457 (11)	−0.0073 (11)	0.0104 (11)	−0.0124 (10)
C18	0.0559 (14)	0.0536 (14)	0.0480 (11)	0.0002 (11)	0.0154 (10)	−0.0012 (10)
C19	0.0603 (14)	0.0398 (13)	0.0595 (13)	0.0064 (10)	0.0123 (11)	−0.0052 (10)
C20	0.0582 (13)	0.0369 (12)	0.0520 (12)	−0.0068 (10)	0.0117 (10)	−0.0091 (10)
Cl1	0.0590 (4)	0.0855 (6)	0.0958 (5)	0.0071 (4)	0.0011 (4)	−0.0167 (4)
N1	0.0455 (9)	0.0267 (8)	0.0378 (8)	−0.0014 (7)	0.0117 (7)	−0.0060 (6)
O1	0.0905 (12)	0.0415 (9)	0.0516 (9)	0.0066 (8)	0.0359 (8)	−0.0060 (7)
O2	0.0520 (9)	0.0478 (9)	0.0635 (9)	−0.0086 (7)	−0.0032 (7)	−0.0030 (7)
O3	0.1027 (14)	0.0303 (9)	0.0566 (9)	0.0054 (8)	0.0096 (9)	−0.0073 (7)
S1	0.0493 (3)	0.0279 (3)	0.0407 (3)	−0.0024 (2)	0.0130 (2)	−0.00589 (19)

### Geometric parameters (Å, °)

**Table:**

C1—C10	1.521 (3)	C10—H10	0.9800
C1—C6	1.536 (3)	C11—H11A	0.9600
C1—C2	1.541 (3)	C11—H11B	0.9600
C1—C7	1.570 (3)	C11—H11C	0.9600
C2—C3	1.532 (3)	C12—O3	1.215 (2)
C2—H2A	0.9700	C12—N1	1.385 (3)
C2—H2B	0.9700	C12—C13	1.521 (3)
C3—C4	1.544 (4)	C13—C15	1.516 (3)
C3—H3A	0.9700	C13—C14	1.537 (3)
C3—H3B	0.9700	C13—H13	0.9800
C4—C5	1.532 (3)	C14—H14A	0.9600
C4—C7	1.544 (3)	C14—H14B	0.9600
C4—H4	0.9800	C14—H14C	0.9600
C5—C6	1.550 (3)	C15—C16	1.386 (3)
C5—H5A	0.9700	C15—C20	1.389 (3)
C5—H5B	0.9700	C16—C17	1.371 (3)
C6—N1	1.481 (2)	C16—H16	0.9300
C6—H6	0.9800	C17—C18	1.377 (3)
C7—C9	1.532 (3)	C17—H17	0.9300
C7—C8	1.533 (3)	C18—C19	1.367 (3)
C8—H8A	0.9600	C18—Cl1	1.745 (2)
C8—H8B	0.9600	C19—C20	1.378 (3)
C8—H8C	0.9600	C19—H19	0.9300
C9—H9A	0.9600	C20—H20	0.9300
C9—H9B	0.9600	N1—S1	1.6915 (15)
C9—H9C	0.9600	O1—S1	1.4241 (15)
C10—C11	1.526 (3)	O2—S1	1.4343 (16)
C10—S1	1.810 (2)		
			
C10—C1—C6	109.61 (15)	C1—C10—C11	115.50 (17)
C10—C1—C2	117.09 (17)	C1—C10—S1	105.23 (13)
C6—C1—C2	104.82 (16)	C11—C10—S1	109.72 (15)
C10—C1—C7	118.83 (16)	C1—C10—H10	108.7
C6—C1—C7	103.48 (16)	C11—C10—H10	108.7
C2—C1—C7	101.34 (16)	S1—C10—H10	108.7
C3—C2—C1	103.66 (19)	C10—C11—H11A	109.5
C3—C2—H2A	111.0	C10—C11—H11B	109.5
C1—C2—H2A	111.0	H11A—C11—H11B	109.5
C3—C2—H2B	111.0	C10—C11—H11C	109.5
C1—C2—H2B	111.0	H11A—C11—H11C	109.5
H2A—C2—H2B	109.0	H11B—C11—H11C	109.5
C2—C3—C4	103.16 (18)	O3—C12—N1	118.56 (18)
C2—C3—H3A	111.1	O3—C12—C13	122.27 (19)
C4—C3—H3A	111.1	N1—C12—C13	119.09 (17)
C2—C3—H3B	111.1	C15—C13—C12	111.07 (16)
C4—C3—H3B	111.1	C15—C13—C14	111.81 (17)
H3A—C3—H3B	109.1	C12—C13—C14	107.14 (18)
C5—C4—C7	102.28 (17)	C15—C13—H13	108.9
C5—C4—C3	107.8 (2)	C12—C13—H13	108.9
C7—C4—C3	102.8 (2)	C14—C13—H13	108.9
C5—C4—H4	114.2	C13—C14—H14A	109.5
C7—C4—H4	114.2	C13—C14—H14B	109.5
C3—C4—H4	114.2	H14A—C14—H14B	109.5
C4—C5—C6	102.59 (19)	C13—C14—H14C	109.5
C4—C5—H5A	111.2	H14A—C14—H14C	109.5
C6—C5—H5A	111.2	H14B—C14—H14C	109.5
C4—C5—H5B	111.2	C16—C15—C20	117.5 (2)
C6—C5—H5B	111.2	C16—C15—C13	120.57 (19)
H5A—C5—H5B	109.2	C20—C15—C13	121.93 (18)
N1—C6—C1	106.71 (14)	C17—C16—C15	121.8 (2)
N1—C6—C5	116.52 (17)	C17—C16—H16	119.1
C1—C6—C5	103.53 (16)	C15—C16—H16	119.1
N1—C6—H6	109.9	C16—C17—C18	119.1 (2)
C1—C6—H6	109.9	C16—C17—H17	120.4
C5—C6—H6	109.9	C18—C17—H17	120.4
C9—C7—C8	106.7 (2)	C19—C18—C17	120.7 (2)
C9—C7—C4	113.4 (2)	C19—C18—Cl1	120.40 (19)
C8—C7—C4	114.6 (2)	C17—C18—Cl1	118.87 (18)
C9—C7—C1	116.57 (17)	C18—C19—C20	119.6 (2)
C8—C7—C1	113.1 (2)	C18—C19—H19	120.2
C4—C7—C1	92.46 (16)	C20—C19—H19	120.2
C7—C8—H8A	109.5	C19—C20—C15	121.2 (2)
C7—C8—H8B	109.5	C19—C20—H20	119.4
H8A—C8—H8B	109.5	C15—C20—H20	119.4
C7—C8—H8C	109.5	C12—N1—C6	120.10 (15)
H8A—C8—H8C	109.5	C12—N1—S1	123.93 (13)
H8B—C8—H8C	109.5	C6—N1—S1	112.09 (12)
C7—C9—H9A	109.5	O1—S1—O2	117.03 (10)
C7—C9—H9B	109.5	O1—S1—N1	109.78 (9)
H9A—C9—H9B	109.5	O2—S1—N1	108.73 (8)
C7—C9—H9C	109.5	O1—S1—C10	111.31 (9)
H9A—C9—H9C	109.5	O2—S1—C10	111.68 (9)
H9B—C9—H9C	109.5	N1—S1—C10	96.30 (8)
			
C10—C1—C2—C3	−168.08 (18)	N1—C12—C13—C15	−120.03 (19)
C6—C1—C2—C3	70.2 (2)	O3—C12—C13—C14	−59.1 (3)
C7—C1—C2—C3	−37.2 (2)	N1—C12—C13—C14	117.6 (2)
C1—C2—C3—C4	2.0 (3)	C12—C13—C15—C16	123.37 (19)
C2—C3—C4—C5	−73.0 (2)	C14—C13—C15—C16	−117.0 (2)
C2—C3—C4—C7	34.6 (2)	C12—C13—C15—C20	−58.3 (2)
C7—C4—C5—C6	−40.9 (2)	C14—C13—C15—C20	61.3 (3)
C3—C4—C5—C6	67.0 (2)	C20—C15—C16—C17	0.9 (3)
C10—C1—C6—N1	34.1 (2)	C13—C15—C16—C17	179.30 (19)
C2—C1—C6—N1	160.52 (16)	C15—C16—C17—C18	1.1 (3)
C7—C1—C6—N1	−93.67 (17)	C16—C17—C18—C19	−1.9 (3)
C10—C1—C6—C5	157.54 (16)	C16—C17—C18—Cl1	176.76 (17)
C2—C1—C6—C5	−75.99 (19)	C17—C18—C19—C20	0.6 (4)
C7—C1—C6—C5	29.83 (19)	Cl1—C18—C19—C20	−178.02 (18)
C4—C5—C6—N1	123.01 (19)	C18—C19—C20—C15	1.5 (3)
C4—C5—C6—C1	6.2 (2)	C16—C15—C20—C19	−2.2 (3)
C5—C4—C7—C9	−63.7 (2)	C13—C15—C20—C19	179.41 (19)
C3—C4—C7—C9	−175.50 (19)	O3—C12—N1—C6	−4.0 (3)
C5—C4—C7—C8	173.5 (2)	C13—C12—N1—C6	179.12 (16)
C3—C4—C7—C8	61.8 (2)	O3—C12—N1—S1	−160.07 (17)
C5—C4—C7—C1	56.7 (2)	C13—C12—N1—S1	23.1 (3)
C3—C4—C7—C1	−55.07 (19)	C1—C6—N1—C12	175.03 (16)
C10—C1—C7—C9	−56.5 (3)	C5—C6—N1—C12	60.0 (2)
C6—C1—C7—C9	65.2 (2)	C1—C6—N1—S1	−26.31 (17)
C2—C1—C7—C9	173.7 (2)	C5—C6—N1—S1	−141.33 (15)
C10—C1—C7—C8	67.7 (2)	C12—N1—S1—O1	−77.31 (17)
C6—C1—C7—C8	−170.59 (19)	C6—N1—S1—O1	124.98 (13)
C2—C1—C7—C8	−62.1 (2)	C12—N1—S1—O2	51.88 (18)
C10—C1—C7—C4	−174.24 (18)	C6—N1—S1—O2	−105.83 (14)
C6—C1—C7—C4	−52.52 (18)	C12—N1—S1—C10	167.33 (16)
C2—C1—C7—C4	55.9 (2)	C6—N1—S1—C10	9.63 (14)
C6—C1—C10—C11	94.4 (2)	C1—C10—S1—O1	−103.83 (14)
C2—C1—C10—C11	−24.8 (3)	C11—C10—S1—O1	131.31 (15)
C7—C1—C10—C11	−147.01 (19)	C1—C10—S1—O2	123.33 (13)
C6—C1—C10—S1	−26.75 (17)	C11—C10—S1—O2	−1.53 (18)
C2—C1—C10—S1	−145.91 (16)	C1—C10—S1—N1	10.28 (13)
C7—C1—C10—S1	91.84 (18)	C11—C10—S1—N1	−114.58 (15)
O3—C12—C13—C15	63.2 (3)		

### Hydrogen-bond geometry (Å, °)

**Table:**

*D*—H···*A*	*D*—H	H···*A*	*D*···*A*	*D*—H···*A*
C20—H20···O3	0.93	2.56	3.038 (3)	112
C13—H13···O1	0.98	2.49	3.278 (2)	137
C9—H9C···N1	0.96	2.52	3.098 (3)	118
C10—H10···O3^i^	0.98	2.54	3.191 (2)	124

Symmetry codes: (i) *x*, *y*+1, *z*.
